# A Multifaceted Programme To Promote Better Living in Icu: Impact On Caregivers' Burnout

**DOI:** 10.1186/2197-425X-3-S1-A40

**Published:** 2015-10-01

**Authors:** A Sader, PM Bertrand, S Moschietto, J Catineau, JP Fosse, JC Orban, N Bèle, L Grech, D Tran, PE Danin, J Dellamonica

**Affiliations:** Nice University Hospital, L'Archet, Medical Intensive Care Unit, Nice, France; Cannes Hospital, Intensive Care Unit, Cannes, France; Avignon Hospital, Intensive Care Unit, Avignon, France; Monaco Hospital, Intensive Care Unit, Monaco, Monaco; Sources Hospital, Intensive Care Unit, Nice, France; Nice University Hospital, St Roch, Intensive Care Unit, Nice, France; Draguignan Hospital, Intensive Care Unit, Draguignan, France; Antibes General Hospital, Intensive Care Unit, Antibes, France; Nice University Hospital, L'Archet, Intensive Care Unit, Nice, France

## Introduction

The French Intensive Care Society issued guidelines *to promotequality of life in ICU* (2009). Though, caregivers' burnout remains a concern.

## Objectives

We studied the relationship between guidelines implementation and caregivers' burnout.

## Methods

The “Comite de Protection des Personnes Sud-Est Mediterranee V” did not consider this research as interventional.

Implementation of the guidelines concerning four areas: (i) unit organization; (ii) patient management; (iii) support for families; (iv) support for caregivers have been studied in the *RIRE* intensive care network.

Meanwhile, we collected anonymously the caregivers' burnout scores using the "Maslach Burnout Inventory".

The relationship between guidelines application and caregivers' burnout have been adressed. Results are median [Interquartile range]

## Results

Seven units (3 academics, 1 medical, 2 surgical and 1 geriatric), representing 64 intensive care beds, were included. 307 caregivers (including 243 women and 64 men, 143 nurses, 95 auxiliaries nurses, 28 physicians, 16 fellows, 7 service agents, 7 medical secretaries, 6 nurses managers, 3 physiotherapists, and 2 psychologists) were evaluated on their burnout.

Median Implementation of the guidelines was 58% [54; 67]. The median implementation rate was 57% [40, 63] for the unit organization, 58% [52, 64] for patient management, 58% [56, 74] for the support for families burden and 58% [54, 64] to support for caregivers. Caregivers reported low to intermediate Malasch score: burnout 12 [7; 19] (low risk), personal achievement 37 [31; 42] (intermediate risk) and depersonalization 5 [2; 10] (low-to-intermediate risk).”

There is a significant association between the guidelines implementation for the support for caregivers and the decrease of burnout score (p < 0.05) and personal accomplishment (p < 0.05). There is a significant association between guidelines implementation for the patient management and decreased depersonalization (p < 0.05).

## Conclusions

Five years after their publication, guidelines on quality of life in the ICU are imperfectly applied. However, they are associated with a decrease in burnout among caregivers, consistent with the literature. Though causality can not be clearly established because of the methodology, this study suggests multimodal interventions may be efficient to improve caregivers satisfaction.
